# Pituitary hypoplasia and growth hormone deficiency in a woman with glycogen storage disease type Ia: a case report

**DOI:** 10.1186/1752-1947-2-210

**Published:** 2008-06-18

**Authors:** Selcuk Dagdelen, Aysegul Atmaca, Ayfer Alikasifoglu, Tomris Erbas

**Affiliations:** 1 Hacettepe University School of Medicine, Department of Endocrinology and Metabolism, Ankara, Turkey; 2 Hacettepe University School of Medicine, Department of Pediatrics, Ankara, Turkey

## Abstract

**Introduction:**

Growth retardation is one of the cardinal manifestations of glycogen storage disease type Ia. It is unclear which component of the growth hormone and/or insulin-like growth factor axis is primarily disrupted, and management of growth impairment in these patients remains controversial. Here we report the first case in the literature where glycogen storage disease type Ia is associated with pituitary hypoplasia and growth hormone deficiency.

**Case presentation:**

A 20-year-old woman with glycogen storage disease type Ia was admitted to our endocrinology department because of growth retardation. Basal and overnight growth hormone sampling at 2-hour intervals demonstrated low levels; however, provocative testing revealed a relatively normal growth hormone response. A hypoplastic anterior pituitary with preserved growth hormone response to provocative testing suggested the possibility of growth hormone neurosecretory dysfunction and/or primary pituitary involvement.

**Conclusion:**

Pituitary hypoplasia may result from growth hormone-releasing hormone deficiency, a condition generally known as growth hormone neurosecretory dysfunction. It is an abnormality with a spontaneous and pulsatile secretion pattern, characterized by short stature, growth retardation and normal serum growth hormone response to provocative testing. However, in the case described in this report, a normal although relatively low growth hormone response during insulin tolerance testing and pituitary hypoplasia suggested that primary pituitary involvement or growth hormone neurosecretory dysfunction may occur in glycogen storage disease type Ia. This is a potential cause of growth failure associated with a lower somatotroph mass, and may explain the variable responsiveness to growth hormone replacement therapy in people with glycogen storage disease.

## Introduction

Growth retardation is one of the cardinal signs and/or complications of glycogen storage disease type Ia (GSDIa). However, the underlying mechanism, and therefore the management of growth impairment, in these patients remains controversial. Hyperlacticacidemia, recurrent hypoglycemia, growth hormone (GH) and/or insulin-like growth factor (IGF) deficiency, GH and/or IGF resistance, decreased insulin and increased cortisol secretions have all been suggested to explain growth retardation in GSDIa [1]. It is not clear, however, at which level the GH-IGF axis is mainly injured. Avoidance of hypoglycemia and hyperlacticacidemia and/or administration of diazoxide or GH replacement therapy have been reported to induce growth, but with variable responses in different patient groups with GSDIa [1-3].

## Case presentation

A 20-year-old woman with GSDIa was referred to our adult outpatient unit because of short stature. She had a birth weight of 3200 g at a gestational age of 35 weeks. She had been diagnosed with GSDIa by glucose-6-phosphatase enzyme assay and quantitative assessment of glycogen content in a liver biopsy specimen at 7 months of age, after displaying classic symptoms and findings consistent with GSDIa including hypoglycemia, lactic acidosis, dyslipidemia, enlarged liver and spleen and nephrocalcinosis, without neutropenia. Recurrent hypoglycemic episodes had been prevented with the use of uncooked cornstarch and frequent feeding over a 20-year follow-up period. However, preprandial hyperlacticacidemia and dyslipidemia persisted despite a lack of hypoglycemia. Initially she grew between -1SD and -2SD. After the age of 7 years growth gradually slowed until it was below -2SD at 9 years of age. She had been evaluated at a pediatric endocrinology department at 14 years of age for growth retardation and delayed puberty. Normal thyroid-stimulating hormone (TSH), follicle-stimulating hormone (FSH), luteinizing hormone (LH) and subnormal GH responses to thyrotropin-releasing hormone (TRH), luteinizing hormone-releasing hormone (LHRH) and GH stimulation (by L-dopa) tests were obtained respectively (Table 1). Menarche was delayed, but occurred spontaneously at the age of 16 years. Liver transplantation had been offered, but refused by the members of her family.

**Table 1:** Endocrine evaluation of the patient at 14 and 20 years of age

Age (years)	14	20
Skeletal age (years)	10	Closed epiphysis
Weight (kg)	25.7	39.8
Height (m)	1.19	1.41
FSH levels (mIU/ml), (Basal/peak response to LHRH stimulation)	0.90/4.90	9.27/11.6
LH levels (mIU/ml), (Basal/peak response to LHRH stimulation)	0.70/9.70	9.57/50.9
Estradiol levels (pg/ml), (Basal/to LHRH stimulated response)	5/20	31.1/315.0
GH levels (ng/ml), (Basal/peak response to L-Dopa stimulation, following estradiol priming)	0.93/6.10	-
GH levels (ng/ml), (Basal/peak response to insulin-induced hypoglycemia when the lowest glucose was 26 mg/dl))	-	0.37/5.18
Overnight GH profile (ng/ml), (Frequent sampling from 10 00 pm to 10 00 am)	-	0.07/0.12/0.37/0.11/0.05/0.24
IGFBP3(ng/ml)	2941	-
Basal IGF1 (ng/ml)	17.5	50.8
TSH levels (mU/ml), (Basal/peak response to TRH stimulation)	4.6/14.5	4.57/21.97
Basal FT4 (pmol/l)	16.7	12.71
Cortisol levels (μg/dl) (Basal/peak response to insulin-induced hypoglycemia)	-	9.9/20.2

Normal values for basal growth hormone (GH): 0 to 7 ng/ml; insulin-like growth factor1 (IGF1), for 12- to 15-year-old girls: 261 to 1096 ng/ml, for 16- to 24-year-old women: 182 to 780 ng/ml; insulin-like growth factor binding protein 3 (IGFBP3), for 7 to 39 year olds: 1250 to 7330 ng/ml; normal GH response to insulin-induced hypoglycemia >5.0 ng/ml; severe GH deficiency in insulin-induced hypoglycemia <3.0 ng/ml; normal GH response to L-Dopa >10 ng/ml.

During her current admission at an adult outpatient unit, her pubertal development was assessed as being complete. She had short stature with a final height of 141 cm (parental target height: 163 cm). She had no hepatic or renal dysfunction. Laboratory tests revealed no hypoglycemia, but hyperlacticacidemia and severe dyslipidemia were seen (low-density lipoprotein = 212 mg/dl, triglycerides = 409 mg/dl, high-density lipoprotein = 27 mg/dl). Functional evaluation of her pituitary gland was performed with provocative testing and showed normal TSH, FSH, LH and GH responses to TRH, LHRH and GH stimulation (by insulin-induced hypoglycemia) tests (Table 1). Morphological evaluation by magnetic resonance imaging (MRI) revealed a hypoplastic adenohypophysis (anterior pituitary height 3 mm) with no abnormality in the neurohypophysis.

Spontaneous overnight GH profiling for 12 hours at 2-hour intervals revealed a mean GH level of 0.16 ng/ml, a spontaneous absolute GH peak level of 0.37 ng/ml and the area under curve value (AUC_GH _= mean profile GH × time) for the night was 115 ng*min/ml (Table 1). Daytime and more frequent overnight GH samplings were offered, but not accepted by the patient.

## Discussion

To the best of our knowledge, hypoplastic adenohypophysis in GSDIa has not been described previously. Melis et al. previously investigated brain MRI findings in patients with GSDI, and showed that 57.1% of the patients had an altered brain MRI pattern [4]. There was no mention in their study of pituitary abnormality or a hypothalamopituitary imaging pattern [4].

Kuemmerle et al. showed that sustained metabolic acidosis causes growth inhibition in rats by decreasing the amplitude and mean mass of GH pulses [5]. As metabolic (hypoxic) injuries of the hypothalamus are also known to cause hypoplasia of the anterior pituitary, the hypoplastic adenohypophysis in the woman described in this case report is considered to be related to growth hormone neurosecretory dysfunction (GHNSD) [6]. GHNSD is an abnormality characterized by short stature, growth retardation and abnormal spontaneous GH secretion despite normal GH response to provocative testing [7]. Our case would formally have satisfied the diagnostic criteria for GHNSD, with a further finding of hypoplastic adenohypophysis. While prepubertal dynamic testing revealed a subnormal GH response, but not severe GH deficiency (that is, stimulated GH response <3 ng/ml), a normal peak GH response was obtained in her re-evaluation using an insulin-tolerance test when she was 20 years old. Such a discrepancy between childhood and adulthood peak GH responses have already been reported in GHNSD [8]. A more frequent sampling than the limited 2-hour intervals undertaken in our case may have detected significant peaks, especially overnight. Spontaneous GH secretion may have been underestimated here.

On the other hand, the existence of GHNSD as a separate entity has been questioned recently in one paper [9]. In this study, which was entirely focused on GH abnormalities following cranial irradiation, it was concluded that a reduced somatotroph reserve might mimic or seemingly present as GHNSD when hypothalamic compensation fails to restore GH secretion in the case of increased demands such as puberty [9]. As growth hormone-releasing hormone (GHRH) also functions as a trophic factor for the pituitary gland, an atrophic or hypoplastic pituitary associated with discordant spontaneous and stimulated GH secretion patterns is generally considered as GHRH deficiency due to GHNSD.

A normal stimulated GH response in an insulin-tolerance test is defined as a peak of above 5.0 ng/ml [10]. Although the stimulated GH level of 5.18 ng/ml in our case was just above the cut-off level, it could still be considered as a relatively low response due to lack of normative data for GSDIa. If so, the reduction in both the spontaneous and stimulated GH secretion, which is proportional to somatotroph volume, may be a reflection of primary pituitary hypoplasia rather than GHNSD.

As described and demonstrated by Darzy et al. in cranial irradiation, the possibility of primary loss in the somatotroph mass, rather than secondary atrophy due to a neurosecretory defect in GHRH secretion, should also be considered, especially in patients with an underlying injury which is potentially harmful to the pituitary gland itself [9]. Although there is a lack of evidence, it is possible that GSDIa may have an associated genetic involvement of the pituitary gland which has not yet been defined.

## Conclusion

We have reported the case of an adult patient with GSDIa in whom GH deficiency was associated with pituitary hypoplasia, and who had a relatively normal GH response to provocative testing, but not classical of GHNSD. In addition to the IGF1 deficiency resulting from primary hepatic involvement, GSDIa also seems to disrupt the hypothalamic-pituitary axis. To clarify whether the pituitary hypoplasia described in this case is a primary occurrence caused by an unknown mechanism, or a secondary event as GHNSD, requires further studies to test spontaneous and stimulated GH secretion patterns in people with GSDIa.

## Abbreviations

FSH: follicle-stimulation hormone; GH: growth hormone; GHNSD: growth hormone neurosecretory dysfunction; GHRH: growth hormone-releasing hormone; GSDIa: glycogen storage disease type Ia; IGF: insulin-like growth factor; LH: luteinizing hormone; LHRH: luteinizing hormone-releasing hormone; MRI: magnetic resonance imaging; TRH: thyrotropin-releasing hormone; TSH: thyroid-stimulating hormone.

## Competing interests

The authors declare that they have no competing interests.

## Consent

Written informed consent was obtained from the patient for publication of this case report and any accompanying images. A copy of the written consent is available for review by the Editor-in-Chief of this journal.

## Authors' contributions

All authors contributed equally to this report. All authors read and approved the final manuscript.
